# A comprehensive review of *Azadirachta indica* (Neem): Exploring proximate composition, bioactive compounds, and prospects for antimicrobial activity

**DOI:** 10.1016/j.bbrep.2026.102730

**Published:** 2026-08-08

**Authors:** Gunasekaran Priyanka, Thirumugam Gowripriya, Bynedi Seshadhri Chinna Mounish, M.N. Trinath, John Bosco Stanislaus, James Prabhanand Bhaskar, Shanmugiah Karutha Pandian, Krishnaswamy Balamurugan

**Affiliations:** a Department of Biotechnology, Alagappa University, Karaikudi, Tamil Nadu, 630 003, India; b Nimyle Neem Research Centre, ITC Life Sciences and Technology Center, Peenya Industrial Area, Bangalore, Karnataka, 560 058, India

## Abstract

Neem, or *Azadirachta indica* A. Juss., is a tree that is both versatile and native to the Indian subcontinent and Southeast Asia. It is frequently employed in traditional medicine to treat skin issues and pyrexia, as well as for environmental protection and insect control. This review reports the multifaceted potential of *A. indica* (neem), a plant that is profoundly rooted in traditional medicine. Neem has high value due to its rich composition of bioactive compounds, and in particular, has a potential for combating several pathogenic microbial communities. There is an increasing necessity to identify novel antimicrobial agents in light of the alarming increase in drug-resistant pathogens. Neem stands out as a promising option, showcasing a wide range of therapeutic benefits that go beyond just treating common issues like fever and skin problems. Its bioactive compounds boast impressive medicinal properties, offering anti-inflammatory, antimicrobial, and immunomodulatory effects that can be incredibly useful for tackling complex health conditions. This review emphasizes the importance of methods of bioactive extraction and biochemical features of neem leaf extracts. There are many bioactive substances such as, triterpenes, flavonoids, and phenolics that contribute to the plant's antibacterial, anti-inflammatory, and antioxidant properties. In addition, this review discusses the immunomodulatory effects and the influence of various extraction solvents on the yield and composition of bioactive compounds.

## Highlights

• Neem contains triterpenes, flavonoids and phenolics with bioactivity.
• Solvent choice affects yield; ethanol/methanol enhance antimicrobial extracts.
• Neem Leaf Glycoprotein shows strong immunomodulatory and anticancer potential.
• Neem extracts are widely used in skin, oral, and wound care products.

## 1 Introduction

In recent years, there has been a strong emphasis on leveraging indigenous knowledge, notably that of ethnobotanists, and researching plants as potential sources of medicine [1,2]. Herbs have also gained commercial relevance as vital ingredients in food, perfumes, and cosmetics, owing to their antioxidant and antibacterial characteristics that act as preservatives [3]. Numerous clinically effective medications for the treatment of acute and chronic illnesses have been developed, thanks in large part to phytochemical substances and their analogs, and studies are still being conducted to find novel therapeutic agents from medicinal plants [4]. According to estimates from the World Health Organization (WHO), over half of the world's population still exclusively uses plants for medical purposes, and almost 80% of people in poorer nations receive their main healthcare only from traditional medicine [5]. The “divine tree” of India, *A. indica*, has been used widely to treat a variety of human ailments, such as pyrexia, ulcers, cancer, diabetes, leprosy, malaria, and skin problems [6]. Antioxidant, hypolipidemic, microbicidal, anti-inflammatory, hepatoprotective, antipyretic, hypoglycemic, insecticidal, antifertility, nematicide, antiulcer, neuroprotective, cardioprotective, and anti-leishmaniasis properties are among the well-known pharmacological qualities of neem tree's extracts [7,8].

Plant secondary metabolites have an important function in antimicrobial detection due to the overuse of antibiotics [9,10]. The widespread and often inappropriate use of antibiotics has accelerated the emergence of antimicrobial resistance (AMR), posing serious threats to human health, the environment, and healthcare systems worldwide [11]. Drug-resistant pathogens have emerged rapidly in both hospital and community settings due to the excessive use of broad-spectrum antibiotics, misuse of reserve drugs for uncomplicated infections, and the easy availability of antibiotics without prescription [12,13]. Consequently, treatment failures have become increasingly common, leading to prolonged hospitalization, increased healthcare costs, and higher rates of morbidity and mortality [14]. Furthermore, the declining effectiveness of first- and second-line antibiotics, coupled with the slow pace of novel antibiotic discovery, has severely limited treatment options for resistant infections [12,14,15]. Recognizing AMR as a major global health challenge, the WHO has emphasized the urgent need for alternative antimicrobial strategies [[16], [17], [18]]. In this context, medicinal plants and their diverse bioactive compounds have gained considerable attention as promising sources of novel antimicrobial agents and adjunct therapeutic molecules capable of combating drug-resistant pathogens [[19], [20], [21]].

In the treatment of common illnesses, plant extracts and phytochemicals with established antibacterial qualities may eventually take the place of antibiotics [19]. Widely grown in tropical and subtropical areas, including India, the neem tree (*A. indica*) is well-known for its many therapeutic benefits and is a major source of therapeutic compounds in conventional herbal medicine [22]. It is a crucial field of research in the battle against antimicrobial resistance because of its adaptability and potential to yield antibacterial compounds.

Among the numerous medicinal plants investigated for their antimicrobial potential, *A. indica* has attracted considerable scientific interest owing to its long history of traditional use and its rich repertoire of pharmacologically active phytochemicals. Its broad therapeutic spectrum, together with increasing evidence supporting its antimicrobial, antioxidant, anti-inflammatory, and immunomodulatory properties, has positioned neem as one of the most promising plant-derived resources for developing alternative therapeutic strategies against drug-resistant pathogens. In traditional folk medicine, almost every part of the neem tree has been used to cure a variety of common illnesses because it has medicinal potential. Fever, skin disorders, leprosy, tuberculosis, intestinal helminthiasis, malaria, and paediatric respiratory infections have all been treated with extracts from the bark, leaves, fruits, seeds, and roots [23,24]. As an efficient dental treatment, neem twigs (datun) are used to brush teeth in traditional Indian customs [25]. The neem tree is one of the most promising, well-established, and possible sources among the many plants that have been investigated for their antibacterial qualities [[19], [20], [21],^25^]. Its widespread application in conventional medicine highlights its potential as a useful tool to develop novel therapeutic molecules. The most important stage when developing novel, potentially effective antibacterial medicines from plants is the biological assessment of herbal items based on their application in traditional folklore. Therapeutic compounds generated from plants may be utilized as a significant substitute, alternative, or supplemental therapy of infectious disorders, according to a number of studies paving up the possibilities for new medication discoveries. To battle these notable issues, more coordinated efforts are needed to study and develop new treatment options. Continued ethnobotanical research is desperately needed to identify and catalogue significant indigenous medicinal plants, their components and explore their potential for the development of antimicrobial components.

Beyond its traditional medicinal applications, different parts of the neem tree have been widely used in public health, agriculture, dentistry, and personal care. This remarkable potential reflects the diversity of pharmacologically active compounds distributed throughout the plant and further emphasizes its value as a rich source of therapeutically essential components. Neem leaves and their paste have long been used to treat allergic skin reactions. They also have antiviral qualities that help prevent chickenpox and smallpox [22,26]. The twigs are frequently used for dental hygiene in traditional medicine [27]. The leaf juice is used as a tonic to help get rid of intestinal worms and increase appetite [28]. Neem is also prized for its hepatoprotective, hypotensive, hypoglycemic, and hypolipidemic qualities, as well as its capacity to regulate fever. The leaf extract is used in Ayurvedic medicine to treat malarial fever and is utilized therapeutically for its antibacterial effect against dental pathogens [26,27,29].

Neem oil has been formulated into effervescent tablets and other eco-friendly formulations for mosquito control [30]. Additionally, skin disorders, cancer, and digestive issues are among the illnesses that are treated with neem and its manufactured products [31]. Flavonoids, saponins, steroids, alkaloids, amino acids, tannins, and phenolic compounds are among the bioactive constituents identified in neem leaves. [28]. Although these substances are relatively hydrophilic, they dissolve better in organic solvents such as alcohol, ketones, and esters. A survey of the literature found that there were few studies on the antioxidant activity, total phenol content, or phytochemical screening of Omani neem species [32]. Nevertheless, there are still questions, such as insufficient data on toxicity levels and inadequate active compound identification using the modern tools and subsequent characterization. Although generations of neem consumption suggests that it is safe, resolving toxicity concerns and making the most of its use require an understanding of its bioactive ingredients, however, the extraction processes and bioactive compound identification requires advanced research strategies.

This review aims to present an in-depth review of the phytochemical screening, total phenol content, and antioxidant activity of crude extracts made from neem, with special reference to its leaves. The study's main goal is to report the biochemical characteristics of the neem extracts, with a focus on investigating different phytocompound extraction techniques. This includes evaluating the efficacy of various extraction processes and their effects on bioactive molecule production and composition. In addition to highlighting their potential as natural antioxidants and antimicrobial agents, this review aims to advance knowledge of the potential health benefits and therapeutic uses of neem leaf extracts by cataloguing previous research and offering insights into the most effective extraction techniques and biochemical properties of these extracts. Given the increasing interest in plant based therapeutic strategies and the immediate need for alternatives to antibiotics, a comprehensive synthesis of the current knowledge on *A. indica* is necessary. Accordingly, this review summarizes the phytochemical composition, extraction strategies, biochemical characteristics, and therapeutic potential of neem, with particular emphasis on its antimicrobial applications and the influence of different extraction methods on the recovery of bioactive compounds.

## 2 Geographical distribution of *A. indica*

Since the bioactive composition and therapeutic potential of herbal plants are often influenced by their geographical origin and environmental parameters, asessing the natural distribution and ecological adaptability of *A. indica* provides an important foundation for interpreting its biological and pharmacological properties. A versatile medicinal plant with a broad spectrum of biological activities, including notable antibacterial properties (Table 1), *A. indica* commonly known as neem has been recognized for over two millennia [2]. Originally native to portions of Southeast Asia and the Indian subcontinent, neem today grows well in tropical and subtropical climates all around [38]. It is very prevalent in India, Sri Lanka, Pakistan, Thailand, Indonesia, Malaysia, and Myanmar; its natural habitat is dry, deciduous woodlands in conjunction with plants including Acacia and *Dalbergia sissoo* [39]. Neem is more common in southern Terai of Nepal. Neem's versatility has helped it to be introduced and established in Australia, the Pacific Islands, South and Central America, the Caribbean, and the Middle East as well as in sub-Saharan Africa. Originally brought in Africa in the early 20th century, neem is now somewhat common in at least 30 nations [40]. Often brought by foresters or popularized by populations familiar with its traditional usage, the tree has also naturalized in Fiji, Mauritius, the Caribbean, and various Central and South American nations [41]. Now growing all over arid subtropical and tropical zones globally, neem is grown in southern Florida in the United States with study plots in southern California and Arizona [42]. Widely prevalent in southern China, South Asia, Southeast Asia, Australia, and has been naturalized in the southern United States, Hawaii, and portions of the Americas and Africa, *Melia azedarach* is a near relative of neem and likewise a member of the Meliaceae family [42]. Though the two species are distinct in both taxonomy and phytochemistry, *M. azedarach*, known by several names including chinaberry, China tree, Texas umbrella tree, and white cedar is occasionally confused with neem due of physical similarities. Though some accounts point to a more general origin spanning arid woodlands throughout South and Southeast Asia, including Pakistan, Sri Lanka, Thailand, Malaysia, and Indonesia, neem is thought to have evolved in the dry forests of Assam and Myanmar. From Kerala in the south to the foothills of the Himalayas, neem is most common today in India, from sea level up to elevations of roughly 700 m [43]. Its global distribution has been motivated by its ecological resilience as well as its appreciated medical, agricultural, and commercial applications.

**Table 1:** Antibacterial effect of neem leaf extract.

S. No	Concentration of extract (ug/ml)	Microbe	Zone of inhibition (diameter in mm)	Methodological details and reference
1	1000	*Staphylococcus aureus*	6.34 ± 0.23	Aqueous leaf extract; paper disc diffusion assay [33]
	2000		6.67 ± 0.17	
	3000		6.79 ± 0.18	
2	1000	*Escherichia coli*	6.26 ± 0.17	
	2000		6.51 ± 0.15	
	3000		6.85 ± 0.25	
3	50% (Methanolic extract)	*Escherichia coli*	17.5 ± 1	Methanolic leaf extract; agar well diffusion assay against clinical and ATCC bacterial isolates [34]
		*K. pneumoniae*	19 ± 1.6	
		*Citrobacter spp.*	22 ± 1	
		*E. faecalis*	16.5 ± 1.8	
		*P. aeruginosa*	21 ± 1	
		*Proteus spp.*	17.5 ± 1.8	
		*S. epidermidis*	15 ± 1.1	
		*S. aureus*	13 ± 0.5	
		*E. coli* ATCC 25922	18 ± 0.4	
		*S. aureus* ATCC 25923	22 ± 0.8	
4	0.05 g/mL	*Escherichia coli*	20	Green-synthesized silver nanoparticles (AgNPs) using neem leaf extract; disc diffusion assay against *E. coli*. [35]
5	50 mg/mL(aqueous extract)	*Escherichia coli*	10.67 ± 0.58	Aqueous and ethanolic leaf extracts (maceration); disc diffusion assay [36]
	50 mg/mL(ethanolic extract)		8.7 ± 0.58	
6	12.5% (ethanolic extract)	*Enterococcus faecalis*	-	Ethanolic leaf extract; microtiter plate antibiofilm assay [37]

## 3 Exploring the bioactive compounds of neem: therapeutic applications and extraction techniques

Numerous studies have demonstrated that *A. indica*, often known as neem, is a storehouse of several bioactive substances, many of which have exceptional pharmacological potential. The group with the greatest medicinal significance among them is triterpenes [44]. Renowned for generating structurally complex triterpenoids with varied bioactivities, *A. indica* has gained major academic attention because of its physiological and economic relevance. A prominent tetranortriterpenoid, azadirachtin, exemplifies this complexity. Its initial experimental synthesis necessitated 71 steps over two decades, but it only obtained a 0.00015% yield, rendering industrial-scale production impractical [45,46]. Recent developments in transcriptomic and genomic investigations have made it possible to find and functionally characterize genes engaged in triterpenoid precursor biosynthesis, especially those controlling limonoid synthesis [46].

Although metabolomic studies have mapped tissue-specific differences in triterpenoid profiles e.g., high azadirachtin levels in young leaves vs nimocinol in mature leaves Neem's genetic and molecular resources remain understudied relative to their well-documented biochemical variety [44]. Omics techniques now offer essential tools to break out the regulatory networks and secondary modification processes (e.g., cytochrome P450-mediated oxidations) driving the production of these pharmacologically important molecules [46]. The most remarkable therapeutic aspect of *A. indica* is the synergistic combination of multiple phytochemicals which is attributed to the beneficial property rather than single bioactive component. Even as triterpenoids, which remained the most extensively investigated group because of their broad pharmacological properties, other secondary metabolites including flavonoids, glycoproteins, alkaloids, and phenolic compounds also potentially contributed significantly to the medicinal benefits of neem. Understanding the biological properties of these bioactives is therefore important for studying the multifaceted therapeutic applications of neem. Neem contains a notable triterpene called nimbin, which has been shown to provide a variety of therapeutic benefits, such as antipyretic, fungicidal, antihistamine, and antiseptic actions. Furthermore, Nimbin is linked to strong antioxidant and anti-inflammatory properties that lessen cellular damage by reducing the generation of reactive oxygen species [47,48].

**Fig. 1:**
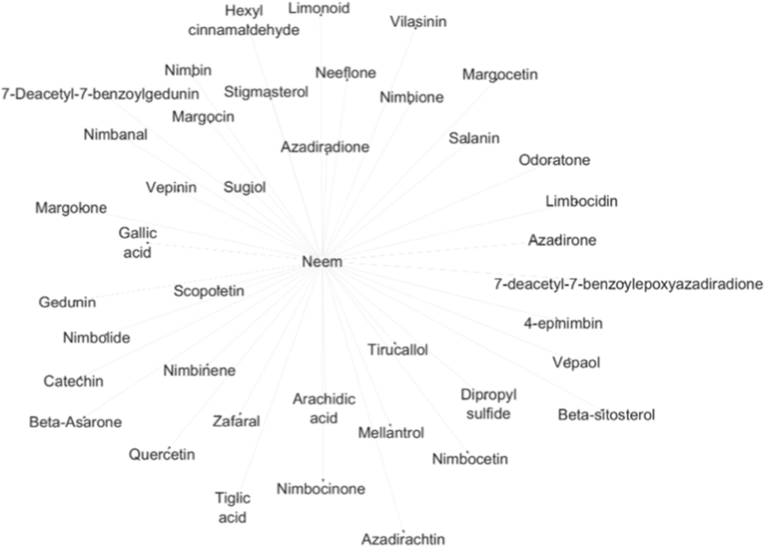
Common metabolites of Neem. **Schematic representation of the major phytochemicals reported from *Azadirachta indica* (neem).** The figure highlights the diverse secondary metabolites identified in different parts of the neem plant, including limonoids, flavonoids, phenolic compounds, phytosterols, terpenoids, fatty acids, and other bioactive constituents.

Flavonoids, another significant class of substances found in neem, are essential for the management of inflammation because they prevent the production of prostaglandins, endoperoxides, and enzymes including phosphodiesterases and protein kinases [49]. The oil extracts from the plant are the most often used type of neem because of their high phytochemical content. Triterpenes, flavonoids, and saponins are found in significant concentrations in these extracts, although catechins and nimbins are found in lesser proportions [[50], [51], [52], [53]]. The extracts contain terpenoids, sterols, limonoids, reducing sugars, alkaloids, tannins, catechins, and gallic acid in addition to these main metabolites [54]. The plant has remained a topic of great interest in both conventional medicine and contemporary pharmacological research due to the adaptability of its bioactive components (Table 2 and Fig. 1).

**Table 2:** Neem compounds and extraction methods.

Neem compound	Source	Biological activity	Extraction method
Azadirachtin	Seed ([55]; [[56], [57], [58]]), Leaf [59], Neem oil [60]	Antimalarial, insecticidal ([55]; [60]), Antiviral [61], Antiinflammatory [59], Biopesticides [57]	Pressurized Liquid Extraction and Maceration with methanol [62], Aqueous extraction [57,61], Ultrasonic extraction with Ethanol and distilled water [59], microwave-assisted extraction with hydroethanolic method [56] and Ultrasonic extraction with acetonitrile [58]
Azadirone, Azadiradione, Epoxyazadiradione	Fruit coat [63,64]	Antitumor, antibacterial	ethyl acetate and water (1:1). Ethyl acetate layer [64,65],
Gallic acid, (−) epicatechin and catechin	Bark	Anti-inflammatory, immunomodulatory	Methanol extract [66,67]
Gedunin	Neem oil, seed kernels	Antimicrobial [68], anti-inflammatory [69]	solated from the limonoid fraction of methanolic extracts of defatted seed kernels by silica gel column chromatography [70]
Nimbin	Bark, Neem oil	Antifeedant	Methanol extract [71], Solid phase extraction [72,73]
Nimbolide	Seed oil, Leaf [59]	Antibacterial [74], anti-inflammatory [59]	Ultrasonic extraction with Ethanol and distilled water [59], Maceration with Ethanol [74]
Nimocinol	Leaves	Insecticidal	[75,76]
Salannin	Seed oil; Leaf [77]	Insecticidal	Maceration based extraction [77,78]

Neem Leaf Glycoprotein (NLGP), a special glycoprotein found in neem leaves, has reported for the strong immunomodulatory effects. In mammalian models, NLGP has demonstrated the capacity to limit tumour growth through the modulation of both systemic and local immunity. By stimulating cytotoxic T cells (CD8^+^) and natural killer (NK) cells, inhibiting regulatory T cells (Tregs), and fostering a type-1 immunological milieu that supports tumour suppression, it improves anticancer immune responses [79,80]. Furthermore, NLGP improves immune cell infiltration into tumours and reduces aberrant vascularization by normalising angiogenesis within the tumour microenvironment [80,81]. Comparative studies of neem leaf extracts have shown that methanolic extracts have higher concentrations of alkaloids, tannins, and flavonoids, whereas aqueous extracts are rich in saponins, tannins, and glycosides [82]. Glycosides including nimbanene, nimbolide, and ascorbic acid, as well as amino acids and proline—which has been related to therapies for neurological illnesses including Parkinson's and Alzheimer's—have also been found in neem leaf extracts [22,82]. Targeting the epithelial-mesenchymal transition (EMT) and enhancing immune responses in preclinical cancer models are two other ways that NLGP has been shown in recent research to delay carcinogenesis [83,84]. The potential of NLGP as a natural, nontoxic immunotherapeutic drug for the treatment of cancer is highlighted by these findings.

Recent research has underscored the effectiveness of plant extracts in the treatment of multidrug-resistant (MDR) bacteria. Ethanol and methanol extracts consistently exhibit more potent antimicrobial activity than hexane and chloroform extracts. Especially, chloroform extracts have exhibited weak antibacterial action, which could be explained by smaller bioactive component concentrations [85,86]. On the other hand, aqueous extracts have been shown useless, presumably because of their poor capacity to extract or preserve enough quantities of antimicrobial compounds. The efficacy of ethanol and methanol in the extraction of aromatic and saturated organic compounds is emphasized by their superior performance, which is important in phytochemical research that is designed to identify new antibacterial agents. This trend highlights the significance of solvent selection in optimizing the extraction of bioactive compounds from plant sources to improve antimicrobial efficacy against MDR bacteria [13,28,32,86]. The antioxidant activity of neem leaf extracts varies depending on the solvent used for extraction. Chloroform extracts have been reported to show stronger antioxidant activity, possibly due to the presence of compounds such as phytol, (2E)-3,7,11,15-tetramethyl-2-hexadecen-1-ol, methyl isoheptadecanoate, methyl 14-methylpentadecanoate, nonacosane, and lineoleoyl chloride. In contrast, methanolic extracts showed comparatively lower antioxidant activity despite containing compounds such as methyl isoheptadecanoate, m-Toluylaldehyde, and methyl 14-methylpentadecanoate, lineoleoyl chloride [28,32,87]. Biologically active substances such butyl palmitate, (2E)-3,7,11,15-tetramethyl-2-hexadecen-1-ol, phytol, methyl petroselinate, hexadecamethyl -cyclooctasiloxane, and other hydrocarbons such as octacosane and nonadecane were discovered to be abundant in hexane-derived extracts. Gallic acid equivalents (GAE) were also used by the group to quantify the overall phenolic content; butanol extracts had the highest phenolic concentration, while hexane extracts had the lowest. Furthermore, dried samples had been subjected to UV analysis to determine the total flavonoid content; the highest quantities of flavonoids were found in methanol extracts, while the lowest levels were found in butanol extracts [28,32,87]. These results demonstrate how extraction techniques affect the antioxidant and bioactive component profiles of neem leaf extracts and highlight the variety of uses they may have in pharmacology and nutrition.

The existence of distinct prenylated flavonoids, such as 5,7,4′-trihydroxy-8-prenylflavanone, 5,4′-dihydroxy-7-methoxy-8-prenylflavanone, 5,7,4′-trihydroxy-3′,8-diprenylflavanone, and 5,7,4′-trihydroxy-3′,5′-diprenylflavanone, has been discovered through the methanolic extraction of neem flowers. These substances, which were absent from the leaves, have shown strong antimutagenic effects against PhIP, Trp-P-I, and Trp-P-II. A wide range of other substances, including diepoxyazadirol, O-methylazadironolide, flowerine, and flowerone, are also present in neem blossoms. Triterpenoids such trichilenone acetate, flavanones like nimbaflavone and 3′-prenylnaringenin, and 4-(2-hydroxyethyl) phenol are other significant components found in the flowers [88].

Extracts from neem have shown strong antibacterial and insecticidal effects. The main ingredient that gives neem its effects on insects is azadirachtin, a complex tetranortriterpenoid limonoid that is present in seeds. Studies have demonstrated that neem leaf ethanol extracts have antibacterial properties against methicillin-sensitive *Staphylococcus aureus* (MSSA) *in vitro* [[89], [90], [91], [92]].

### 3.1 Phenolic compounds in neem leaf extract: antioxidant properties and health benefits

Phenolic chemicals are phytocomponents with redox characteristics that contribute to antioxidant action [93]. These substances are abundant in plants and have potent antioxidant properties through their roles as metal ion chelating agents, hydrogen donors, and reducing agents. The qualitative and quantitative characteristics of the polyphenol chemicals in neem leaf powder have been examined in a number of research studies. According to reports, the total phenolic content of neem leaves is 70 mg GAE (gallic acid equivalents)/g, and the total flavonoid content is 119 mg QE (quercetin equivalents)/g [94]. Furthermore, 10.80 mg g^−1^ (1.08%) of total phenolic content is found in neem leaf flour that has been dried under ideal conditions [95]. Because of their antioxidant qualities, phenolic compounds have attracted a lot of attention for their potential to improve health, especially in the fight against cancer and neurological illnesses. Higher plants are rich in flavonoids, which also act as antioxidants to guard against free radicals, which can damage tissues and cells. According to research, flavonoids may promote general cardiovascular health, including the integrity of artery walls, and prevent the proliferation of human cancer cells [96]. Collectively, these findings highlight phenolic compounds as one of the major contributors to the antioxidant activity of neem.

### 3.2 Saponins in neem leaf extract: functional and health-promoting properties

Glycosidic substances called saponins can often be found in food [97]. Because of their capacity to foam and emulsify, these naturally occurring chemicals are commonly used in food science to improve the texture and stability of beverages such as soft drinks and beers [98]. Saponins have potential health benefits, such as cholesterol-lowering, anticancer, and antioxidant properties, in addition to their functional responsibilities. The amount of saponins in neem leaves varies depending on the plant variety, growth environment, and extraction technique. According to studies, the saponin content of neem leaves ranges from 2.4% to 10% in aqueous extraction, depending on agronomic conditions [96,99,100]. Together, these observations demonstrate that saponins contribute not only to the functional properties of neem but also to its diverse biological activities.

### 3.3 Neem leaf glycoprotein: a bioactive component with antitumor and emulsifying properties

Glycoproteins are proteins made up of oligosaccharide carbohydrate chains connected to amino acid side chains [101]. As thickening agents, stabilisers, and emulsifiers, they are widely used in the food sector to improve the texture, stability, and shelf life of a variety of products [102]. Specifically, in culinary products like sauces, dressings, and drinks, glycoproteins aid in the combination and stabilisation of immiscible components like water and oil [103]. Neem leaf glycoprotein (NLGP), which are abundant in neem tree leaves, have been shown to be effective in preventing tumour growth by modifying both systemic and local immunity [81,104,105]. According to recent studies, neem leaves are a good source of glycoproteins that are safe for bodily processes and may be useful in the treatment of cancer [106]. These findings emphasize the unique immunomodulatory potential of NLGP and support its future therapeutic development.

### 3.4 Neem leaf alkaloids: functional roles in plant defence and human health

Alkaloids are naturally occurring nitrogen compounds found in plants, fungi, and animals [107]. Alkaloids, which are found in around 25% of plant species and are well-known for their physiological and pharmacological properties, are essential to plant survival because they function as organic herbicides. They are important for a number of uses, such as medicines, chemicals, and supplements in human medicine. Alkaloids that activate the human brain include theobromine in cacao and caffeine in coffee [108]. Alkaloids have alkaline qualities and can block a variety of pharmacological actions; some of them are used to treat cancer and act as respiratory and cardiac stimulants [109]. The presence of alkaloids in methanol and ethanol extracts from neem leaves has been evidenced by phytochemical studies [82,110,111]. Aslam et al. [96] stated that the alkaloids in powdered neem leaf are 4.10 g/100 g (4.10% dry matter). On the other hand, pulverized neem leaf had a greater total alkaloid content of 10.67 g/100 g (or 10.67%), according to another study [100]. The neem trees' cultivation in different agro-ecological zones may be the cause of this disparity in alkaloid levels [112]. Although present in varying concentrations, alkaloids further expand the pharmacological repertoire of neem.

### 3.5 Carotenoid content in neem leaf extract: nutritional benefits and health implications

Fruits and vegetables that are yellow, orange, or red contain carotenoids [113]. Pulverized neem leaf has been shown to contain 110,000 μg/100, or 1.1%, of carotenoid concentration [114]. Neem leaf extract has a significant carotenoid level (1995 μg/100g, or 0.019%) [115]. Carotenoids, which give many fruits and vegetables their appealing colour, are among the phytochemical components thought to lower the chance of acquiring certain degenerative diseases [116]. According to Del Campo et al. [117], these carotenoids can be employed as colouring agents, provitamin A in food and feed, cosmetic additives, and in the manufacturing of multivitamins. The nutritional and antioxidant properties of carotenoids complement the overall therapeutic potential of neem leaves.

### 3.6 Neem leaf extract: a rich source of ascorbic acid

Ascorbic acid and amino acids, among other substances, are thought to be abundant in neem leaves and may assist plants minimise the negative impacts of water scarcity. Often vitamin C, is referred to as ascorbic acid, a water-soluble vitamin that is naturally present in a wide variety of foods. It is a potent antioxidant that reacts quickly with peroxyl and superoxide radicals. Along with vitamins E, A, B1, and B2, neem leaves are especially rich in ascorbic acid and amino acid [22,118]. According to Keta et al. [119], Keta et al. [120], the amount of vitamin C in neem leaves is 0.31g/100 g. Ascorbic acid plays a vital role in collagen synthesis and is essential for iron absorption, as well as the metabolism of certain proteins and folic acid. It also keeps other vitamins from oxidising, aids in the metabolism of calcium and amino acids, stops internal bleeding, fortifies blood vessels, keeps bones and teeth healthy, and speeds up the healing of burns and wounds [121].

## 4 Optimizing solvent selection for efficient plant extraction: principles, guidelines, and safety considerations

Plant extracts are significant resources in a variety of industries, such as pharmaceuticals, cosmetics, food production, personal care production and herbal medicine. The quality and efficiency of the resultant product depend much on the extraction technique, especially in terms of the solvent selection. Strategically selecting suitable solvents is essential for the extraction of bioactive compounds from plant materials [122,123]. Safety standards, cost-effectiveness, and environmental sustainability are all ensured by this selection, which also affects extraction yield and purity. The majority of extraction processes are governed by the principle “like dissolves like,” which states that polar solvents extract polar compounds and non-polar solvents extract lipophilic substances [124]. While intermediate polarity is obtained with acetone and dichloromethane, water, ethanol, and methanol are the most often used polar solvents in plant extraction methods [125]. Effective extractives for collecting non-polar components are hexane, ether, and chloroform. Sometimes called the traditional word “menstruum,” these solvents must be precisely matched to target molecules based on their chemical characteristics [124].

In order to optimize extraction efficiency, it is imperative to comprehend the chemical composition of both the plant material and the potential solvents [124]. Another critical parameter during extraction operations is temperature, as thermolabile compounds may be compromised by high temperatures [126]. The sensitivity of vitamin C and various polyphenolic substances to thermal degradation is particularly evident in their extraction with polar solvents [127,128]. Polar solvents include water, methanol, and ethanol efficiently isolate polar molecules; non-polar solvents including hexane and dichloromethane selectively capture lipophilic components [124]. The extraction specificity follows predictable trends. The traditional liquid-liquid extraction protocols typically involve the use of carefully selected solvent pairings, such as water-ether, water-chloroform, water-hexane, or water-dichloromethane. Water is a prominent component of these protocols due to its distinct polarity and ability to phase separate with organic solvents.

Valuable insights into the efficacy of solvents are provided by empirical research. According to Ref. [129], the most effective extraction efficiency and gallic acid concentration were achieved by water extracts of *Labisia pumila*, with ethanol, ethyl acetate, and hexane extracts following in that order. A recent study [130] has confirmed the superiority of ethanol in the extraction of chlorogenic acid and flavonoid compounds. Aqueous acetone is the most effective method for extracting higher molecular weight flavanols, and methanol is the preferred method for extracting lower molecular weight polyphenols. In commercial applications, the selection of solvents is significantly influenced by safety profiles. Ethanol is particularly well-suited for food-grade applications and environmentally conscious extraction processes due to its reduced toxicity profile, which distinguishes it from acetone, methanol, and other organic solvents. In contrast, the implementation of solvents such as acetonitrile, chloroform, hexane, and methanol in pharmaceutical products necessitates caution, as they have established concentration limits of 400, 60, 290, and 3000 ppm, respectively. Therefore, selecting an appropriate extraction solvent is fundamental for maximizing phytochemical recovery and ensuring the therapeutic efficacy of neem-derived products.

## 5 Advancing *in vitro* and *in vivo* research on neem extracts for safe antimicrobial applications

Extensive study on the antibacterial qualities of neem has resulted from the need to find novel therapeutic agents due to the rising frequency of antibiotic-resistant bacterial infections [131]. The successful applications of neem in the food industry and its historic use in dental hygiene lend credence to this approach. Furthermore, the capacity of pathogenic bacteria to build biofilms has attracted a lot of attention since these formations lead to increased resistance to antibacterial agents, which makes treating infections extremely difficult [5,23]. Few practical methods for eliminating biofilms have been devised, despite the well-established significance of biofilm-associated illnesses in human disorders [132]. Neem is a good option for medication discovery, nevertheless, as encouraging data indicates that it regularly outperforms a wide range of other herbal extracts in preventing bacterial growth and focussing on cells that have formed biofilms. Neem has been shown in studies to have strong antibacterial activity against a variety of strains, including Gram-negative uropathogens that produce metallo-beta-lactamase (MBL) and extended-spectrum beta-lactamase (ESBL) [133,134]. It has also been shown to be effective in lowering surgical site infections when applied to coated sutures [132,135,136]. These results demonstrate neem's promise as a source for creating new antibacterial medicines, especially against illnesses linked to biofilms and microorganisms resistant to antibiotics.

*In vitro* techniques like as broth dilution, disc or agar diffusion, and agar overlay assays are frequently used to assess the antibacterial properties of neem extracts and their phytochemicals [86]. These methods aid in finding out each treatment's minimum bactericidal concentration (MBC) and minimum inhibitory concentration (MIC). In order to more closely mimic human infections and illnesses, a number of *in vivo* models have also been used. In these models, neem oil-related medications are usually administered orally or gastrically, as well as intraperitoneally or intravenously to mice, rats, guinea pigs, and rabbits [137,138].

Although *in vitro* antimicrobial assays provide valuable preliminary evidence for the biological activity of neem extracts, validation in living organisms is essential to understand efficacy, toxicity, and mechanisms of action under physiologically relevant conditions. Consequently, several *in vivo* models have been employed to evaluate the therapeutic potential of neem-derived compounds. Patel et al. utilized *Caenorhabditis elegans* as a living model to evaluate how effective Herboheal® (License No. GA/1616; SRISTI Organization, Ahmedabad, Gujarat, India), a wound healing formulation with a blend of various herbs including neem, is against infections caused by *Staphylococcus aureus* [139]. When *S. aureus* was pre-treated with Herboheal®, there was a notable decrease in its ability to harm *C. elegans*, suggesting that the formulation helps reduce the bacteria's virulence by influencing Quorum Sensing (QS) and genes linked to its harmful effects. Analyzing the transcriptome showed a downregulation of important *S. aureus* genes that play roles in hemolysis, virulence, and QS (such as the agr system), all of which are vital for damaging host tissues and dodging the immune system. This reduction in virulence factors was associated with improved survival rates of *C. elegans*, supporting the traditional use of Herboheal® in treating wounds and emphasizing its promise as a potential anti-infective solution against antibiotic-resistant strains like *S. aureus* [139].

A recent study evaluating into the anthelmintic properties of neem, in combination with *Andrographis paniculata* and *Litsea elliptica*. Studies used *C. elegans* as a model organism to tackle the issue of multi-drug resistance in helminth control [140,141]. To evaluate the anthelmintic effectiveness, *C. elegans* are exposed to various concentrations of these extracts and monitored head thrashing activity and mortality rates. The results showed that both the water and ethanol extracts of *A. indica* caused significant mortality, highlighting its potential as a natural alternative to synthetic drugs for treating parasitic infections [140,141].

Similarly, vertebrate models have been employed to evaluate additional pharmacological properties of neem beyond antimicrobial activity. Recent studies have shed light on the exciting potential of neem fruit's ethanolic extract as a strong pain inhibitor using zebrafish. The study showed that this extract has notable antinociceptive effects in acute pain models, suggesting that it works by influencing the opioid system, NMDA (N-methyl-d-aspartate) receptors, and ASIC (Acid Sensing Ion Channels) [142]. These results not only add to the scientific evidence supporting the pain-relieving properties of *A. indica* fruit but also highlight the need for more research to identify and understand its active components. Overall, this work positions neem bioactives as a promising candidate for creating new plant-based medicines aimed at pain management [142].

According to published animal research, the particular plant component used, the extraction solvent, the administration route, and the species used in the model all have a substantial impact on the acute toxicity of neem extracts [138]. For example, mice did not die when given an oral dose of ethanolic neem leaf extract at levels lower than 2000 mg/kg body weight [143]. On the other hand, rats given ethanolic extract from neem stem bark at dosages ranging from 50 to 200 mg/kg experienced changes in biochemical indicators of toxicity and perhaps experienced negative impacts on organ function [144].

While these studies collectively demonstrate promising biological activities, safety evaluation remains essential before clinical translation. A human study by Bandyopadhyay et al. [145], found that a lyophilised aqueous neem bark extract administered at doses of 30–60 mg twice daily for 10 weeks showed therapeutic potential for managing gastric hypersecretion and gastro-oesophageal and gastroduodenal ulcers, with no discernible effects on blood parameters indicating organ toxicity. 156 adults and 110 children reported no significant negative effects following a year of external exposure to 1% neem oil. Notably, Arsene et al. (2021) showed that ethanolic extracts of neem leaves and seeds showed higher toxicity levels than their aqueous counterparts, proving that the *Galleria mellonella* (wax moth larva) model is useful for evaluating acute *in vivo* toxicity of medicinal plants.

The US Environmental Protection Agency has declared that cold-pressed neem oil has “no unreasonable adverse effects to the US population and the environment,” even though the *in vivo* data need to be further developed before neem oil extracts and phytochemicals can be used clinically [146]. While raw materials and pure phytochemicals from the tree have very low levels of toxicity, according to the Boeke et al., non-aqueous extracts of neem are the most toxic neem-based products for humans when consumed orally. The estimated safe dose (ESD) is quite low, ranging from just 0.002 to 12.5 μg per kilogram of body weight each day. The most notable toxic effects seen with sub-acute or chronic exposure to all neem preparations, including those non-aqueous extracts, appear to be reversible impacts on the reproduction of both male and female mammals [147]. Standardizing extraction techniques and administration protocols should be a key goal for future antimicrobial testing of neem oil and its derivatives, given the current data compiled here. Researchers need more comparative report and data on different solvent-based extraction bioactives from *A. indica's* for their possible uses.

A number of critical research directions must be prioritized in order to advance the development of therapies based on *A. indica* (neem) for human use. Future research should concentrate on clarifying the processes of action of neem-derived molecules, especially against drug-resistant bacteria. Important areas of research include phytochemical optimization, whereby isolated neem compounds or their derivatives are tested as standalone antimicrobial agents or adjuncts to current treatments with dosing and safety protocols. A rigorous preclinical and clinical trial is needed to establish toxicity profiles and effective *in vivo* dosages for specific phytochemicals compared to crude extracts. A broad-spectrum screening with standard methods for evaluating antimicrobial potential across diverse medicinal plant extracts is the need of the hour to address emerging infectious threats. Meaningful comparisons and hastening of finding will be made possible by standardizing extraction techniques and phytochemical characterization across different research fields. The extensive research framework has to be developed for neem to serve its bioactive components as a paradigm for the investigation of other traditional medicinal plants, providing a means to combat antimicrobial resistance while utilizing the global phytochemical diversity.

## 6 Multifunctional applications of neem in health and personal care

Neem oil is abundant in bioactive compounds, with over 100 identified constituents, with triterpenoid limonoids being the most important [148]. Though, azadirachtin is the main active chemical, the other important ingredients of neem oil are meliantriol, nimbin, nimbidin, nimbinin, nimbolides, salannin, and fatty acids including oleic, stearic, and palmitic acids [148]. The main source of commercial oil extraction, neem seeds have the highest azadirachtin content. Although other tree parts such as leaves, barkhave less azadirachtin, they are nonetheless used in oil manufacture. By means of artificial inoculation, research shows that arbuscular mycorrhizal fungus can increase azadirachtin content in seeds, therefore providing a possible means to maximize bioactive component yields [149,150]. With seed-derived oil remaining the most powerful for agronomic and medical uses due of its high limonoid concentration, neem oil is extracted using several procedures including cold pressing and solvent extraction. Neem oil is a great natural resource for handling a variety of skin, scalp, dental, and personal care needs because of its adaptability and broad spectrum of bioactive components [151,152]. Overall, the modern scientific study as well as conventional usage informations collectively helping to justify its effectiveness on the following applications.

➢ Anti-Aging: Neem oil helps old scars heal, increases skin suppleness, and stimulates collagen creation. Its strong antioxidant profile which includes vitamin E and vital fatty acids helps combat free radicals and slow the outward indicators of aging, therefore producing softer, more flexible skin [153].
➢ *Acne* Treatment: Neem oil reduces the ability of *Propionibacterium acnes* to generate reactive oxygen species (ROS) and pro-inflammatory cytokines while treating acne. Demonstrated *in vitro*, its antibacterial action consists in upsetting the formation of bacterial cell membrane, therefore preventing the spread of acne-causing germs. Key active components like margolone, nimbolide, and mahmoodin add to its antibacterial properties; gedunin and cyclic trisulphide have antifungal action. A complete treatment for acne-prone skin, neem oil also helps control sebum production, clear pores, lower inflammation, and hasten healing of acne scars [154].
➢ Oral Care: Neem extract is employed in dental lubricants and oral hygiene products to inhibit *Streptococcus mutans*, the bacterium that causes tooth decay, treat gingivitis and periodontitis, and reduce plaque. More successfully than conventional mouthwashes including chlorhexidine gluconate, clinical trials reveal neem-based dental gels can lower plaque and bacterial load ([155]; [22]).
➢ Anti-Dandruff: Neem oil's antibacterial, antifungal, and anti-inflammatory qualities help it be useful against scalp irritation and dandruff. It helps general scalp health, lessens flaking, and calms irritation [156].
➢ Pediculosis (Head Lice) Treatment: Shampoos using neem seed extract have shown better effectiveness against head lice than formulations based on permethrin, therefore providing a natural alternative for lice management [157].
➢ Wound Healing: Neem oil facilitates wound healing by fostering tissue repair and reducing inflammation, which is facilitated by the presence of tannins and other bioactive compounds [22].
➢ Health and Personal Care Products: Neem oil finds application in a variety of goods, including shampoos, conditioners, toothpastes, soaps, skin cleansers, nail oils and polishes. Its general antibacterial and therapeutic qualities make it a useful component in these compositions [109,158,159].
➢ Skin Care: Neem oil is included in topical treatments for lice and scabies as well as in soaps [160].
➢ In most cases, neem has been demonstrated to cure scabies when mixed with *Curcuma longa* (turmeric) in a paste over 3 to 15 days; no side effects have been recorded. Neem helps also with wound healing, dry psoriasis, and diabetic foot ulcers.

Neem oil has been shown to inhibit tyrosinase activity by 47%, which contributes to a skin-brightening effect. However, it does not contain anti-wrinkle properties and is most appropriate for youthful skin [153,160,161].

## 7 Metabolomics-driven insights into neem: advancing therapeutic applications through comprehensive profiling

Metabolomics, which concentrates on the comprehensive profiling of small molecules, is distinguished by its ability to capture the final products of gene expression, thereby offering the most direct and authentic representation of the organism's physiological state. Targeting individual substances, wide profiling, metabolic fingerprinting, or full-scale metabolomics-each providing different degrees of information and discovery potential metabolic studies can be pursued [162,163]. As sensitive indicators for plant-microbe dynamics, these approaches help scientists to link microbial community composition and function to particular plant phenotypes and environmental interactions [164]. The high-throughput and scalability of metabolomics have transformed natural product discovery and bioprospecting of neem metabolites, therefore enabling the identification of bioactive marker metabolites prior to committing to labor-intensive downstream activities [165]. Often used with separation methods like gas or liquid chromatography, analytical platforms such nuclear magnetic resonance (NMR) and mass spectrometry (MS) are fundamental to these studies and each have special advantages for metabolite detection and quantification [163] (Supplementary Table 1). These advances have significantly expanded the application of metabolomics from simple metabolite identification to mechanistic investigations of neem bioactivity, enabling a more comprehensive understanding of its therapeutic potential.

By means of high-throughput metabolomic analyses, we can pinpoint and define the particular metabolites linked to anti-diabetic, anti-hypertensive, anti-hyperlipidemic, and antioxidant activities-triterpenoids, flavonoids, and polyphenols that account for the therapeutic effects of neem [[166], [167], [168], [169], [170], [171]]. Standardizing and quality control of neem extracts is made possible by this thorough metabolic fingerprinting, therefore guaranteeing constant efficacy and safety in therapeutic application [166,167]. By identifying active constituents and their impact on molecular pathways including Nrf2-mediated antioxidant defense, GLUT4 expression, and inhibition of glucosidases, metabolomic studies have helped clarify the mechanisms by which neem modulates metabolic syndrome parameters—such as reducing blood glucose, improving insulin sensitivity, regulating lipid profiles, and enhancing endothelial function [22,172,173]. Moreover, standardized neem extracts have shown in clinical investigations supported by metabolomic data to greatly enhance glycemic management, lower inflammation and oxidative stress, and lower cardiovascular risk indicators in patients with metabolic syndrome [22,166,167,172,173]. All things considered, the metabolomic method not only speeds the identification of new therapeutic molecules from neem but also supports the development of evidence-based, focused treatments for certain chronic diseases.

## 8 Future perspectives

*A. indica* (neem) is acknowledged as a medicinal tree of exceptional value and a significant source of azadirachtin, which is known for its biological properties. Beyond azadirachtin, Neem synthesizes a broad spectrum of biologically active molecules having demonstrated therapeutic advantages. Although a great deal of study has been done on its enzymatic routes, the downstream genes involved in azadirachtin production remain unknown, which emphasizes the need of more study with integrated omics techniques. By employing genomics, transcriptomics, proteomics, and metabolomics, researchers can develop a comprehensive comprehension of the biosynthetic pathways that underlie a variety of alkaloids and their derivatives in *A. indica*. Identification and separation of bioactive chemicals necessary for industrial and medicinal uses depend on this method. Omics technologies promise to map biosynthetic routes, identify new bioactive compounds, assess genetic diversity, hasten functional gene discovery, direct breeding programs, and use systems biology approaches. Such developments highlight the great potential of omics-driven research in releasing the full possibilities of *A. indica*, hence greatly improving the development of Neem-based solutions across home and personal care products with a broad area covering medical, agricultural, and industrial sectors. Despite the promising therapeutic potential of *A. indica*, several challenges need to be addressed before its bioactive compounds can be translated into clinical applications. Variations in phytochemical composition due to geographical origin, environmental conditions, and extraction methods can affect the consistency and efficacy of neem-based products. In addition, the lack of standardized extraction protocols, limited clinical studies, and insufficient information on pharmacokinetics and long-term safety remain important limitations. Future research should focus on standardization, quality control, and well-designed clinical studies to facilitate the development of safe, effective, and evidence-based neem-derived therapeutics.

## CRediT authorship contribution statement

**Gunasekaran Priyanka:** Conceptualization, Data curation, Formal analysis, Investigation, Methodology, Writing – original draft. **Thirumugam Gowripriya:** Conceptualization, Data curation, Formal analysis, Investigation, Methodology, Writing – original draft. **Bynedi Seshadhri Chinna Mounish:** Formal analysis, Investigation. **M.N. Trinath:** Conceptualization, Investigation, Methodology, Validation. **John Bosco Stanislaus:** Conceptualization, Data curation, Methodology, Validation. **James Prabhanand Bhaskar:** Conceptualization, Data curation, Investigation, Methodology, Writing – review & editing. **Shanmugiah Karutha Pandian:** Conceptualization, Data curation, Methodology, Writing – review & editing. **Krishnaswamy Balamurugan:** Conceptualization, Data curation, Methodology, Supervision, Writing – review & editing.

## Declaration of competing interest

The authors declare that they have no known competing financial interests or personal relationships that could have appeared to influence the work reported in this paper.

## Data Availability

Data will be made available on request.
