# Supplementary material for: A comprehensive review of Azadirachta indica (Neem): Exploring proximate composition, bioactive compounds, and prospects for antimicrobial activity
Source: Biochem Biophys Rep. 2026 Aug 8;47:102730. doi: 10.1016/j.bbrep.2026.102730 (PMC13476478; doi:10.1016/j.bbrep.2026.102730)
Supplement: Supplementary file 1 [file mmc1.doc]

Supplementary Table 1: Extraction methods along with identified metabolites in neem extracts

| **S.No** | **Reference** | **Part Used** | **Method for extraction** | **Solvent used** | **Method for**  **compound detection** | **Compounds detected** | **Library used** |
| --- | --- | --- | --- | --- | --- | --- | --- |
| 1. | Dhillon *et al.*,2024 | Leaf Powder | Sun dried | - | FTIR | - Nimbolide, - Azadirachtolide, - β- sitosterol, - Quercetin | - MATLAB Software was used for the spectral analysis |
| 2. | Mohammad *et al.*,2024 | Seed | Soxhlet extraction | 6:4 Hexane: Ethanol | GCMS | - Octamethyl cyclotetrasiloxane - Decamethyl cyclopentasiloxane - 3-(Isopropyl)-1,5-cyclohexadiene-1-methanol - (2,6,6-Trimethyl-cyclohexa-1,3-dienyl)-pent-3-en-2-one - Methyl dodecanoate - 1,1,1,2,2,5,5,6,6-nonafluorooctane - 5-cyano-6-ethoxy-2-methyl-4-(4-pyridyl)- 4H-pyrane-3-carboxamide - 3-cyclohexene-1-methanol - Cis-p-mentha-2,5-dien-7-ol - Methyl tetradecanoate - (6-bromo-3-cyclohexen-1-yl)-Propanedinitrile - 4-(1-methylethyl)-1,5-   cyclohexadiene-1-methanol   - Methyl 14-methylpentadecanoate - Methyl 14-methylpentadecanoate - (Z)-9-octadecenoic acid, - methyl ester (Methyl oleate) - Methyl 6-octadecenoate - Methyl 9-cis,11-trans-octadecadienoate - 3-tetradecen-1-ol acetate - cis-13-Eicosenoic acid, methyl ester (Methyl cis-13-eicosenoate) | - NIST/EPA/ NIH mass spectral library Version 2.2, along with   the NIST mass  spectral search program. |

|  |  |  |  |  |  | - Methyl eicosanoate - N-butyl-, cis-4-Cyclohexene-1,2-dicarboximide - 2-butyl-4,5,6,7-tetrahydro-1H-Isoindole-1,3(2H)-dione |  |
| --- | --- | --- | --- | --- | --- | --- | --- |
| 3. | Alhaithloul, *et al.*,2023 | Leaf Powder | Maceration | 30:70 Hydro-methanol | GCMS | - 3-(tert-butyl)-4,5,6,7-tetrahydro-1H-indole, - 6-methyl-9H-carbazole-3,4-diyl)bis((4   chlorophenyl)methanone,   - (2R)-1,3,8-trimethyl-4-propyl-5-ethyl-2-(1-hydroxyethyl)-7-methoxycardonylethyl-6-g-methylenecarbonyl-porphyrin, - (E)-3,7-dimethylocta-2,6-dienal, - 7-methoxy-3,4-dihydro-2H-spiro[naphthalene-1,2'-[1,3]dithiolane], - 4,5-dimethylheptan-3-ol, - Methyl oleate, Methyl (E)-octadec-13-enoate, - Methyl docosanoate, bis(2-ethylhexyl) adipate, - Citral | - WILEY 09   - NIST 14 |
| 4. | Almaghamsi *et al.*, 2023 | Leaf powder | Maceration | Methanol, Ethanol and  Distilled water | HPLC | - Ferulic acid, - 4-hydroxy - benzoic acid | - Based on Retention time |
| 5. | Mayavel *et al.*, 2023 | Seed Oil | Soxhlet Extraction | Petroleum ether | Statistical analysis |  |  |
| 6. | Rajasekharan *et al.*, 2023 | Leaf | Soxhlet Extraction | 95% Ethanol | Thin flim formation | - |  |
| 7. | Jahan & Arju, 2022 | Leaf | Heating at 100°C | Aqueous | - | - |  |
| 8. | Sarkar *et al.*, 2022 | Bark | Maceration | Methanol | - | - |  |

| 9. | Mehta *et al.*, 2022 | Leaf Extract | Maceration | 95% Ethanol | Antimicrobial assay | - |  |
| --- | --- | --- | --- | --- | --- | --- | --- |
| 10. | Seriana *et al.*, 2021 | Leaf Extract | Maceration | 70% Ethanol | GCMS | - 2-isopropenyl-1-methyl-4(1-   methylethlidne) vinylcyclohexane),   - neophytadiene, - 1,2-benzenediccarboxylic acid,   dibutyl ester,   - hexadecanoic acid,   ethyl ester,   - n-hexadecanoic acid, - azulene, 7-ethyl-1,4-dimethyl-, - (2e)-3,7,11,15-tetramethyl-2-hexadecen-1-ol, - phytol, - 1,4,6,8-tetramethyl-(1,3a-(13c)-azulene, - linolenic acid, - 2’,5’-dimethoxy-2-   biphenylcarboxylic acid,   - nanocosane, - stigmastan-3,5-diene, - octacosane, - vitamin e, - 4-methoxy-2-butyn-1-ol, - pentacosane, - trioctylphodlhine, - octadecanoic acid, - cycloprone,   1,1,2,2-tetramethyl-3-(2-phenylethenylideno)-,   - gamma. -elemena, - germacrene b, - 7-ethyl-9,9-dimethylspiro (4.5) dec-7ene-1,4 dione | - |

| 11. | Rangiah *et al.*, 2016 | Leaf Extract | - Sonication - Incubation | Methanol, acetonitrile, acetone,  ethyl acetate, Methyl-tert-butyl ether) | UHPLC-MS/SRM | - Azadirachtin A - Nimbin - Salanin - Epoxy or hydroxy azadiradione | - Based on standard curve (the area ratio versus the   concentration of  analyte on the column) |
| --- | --- | --- | --- | --- | --- | --- | --- |
|  |  |  |  |  |  | - Azadirachtin A - Nimbin - Salanin - Azadiradione - Epoxy or hydroxy azadiradione |  |
|  |  | Seed Extract |  |  |  |  |  |
|  |  |  |  |  |  | - Azadirachtin A - Nimbin - Salanin - Epoxy or hydroxy azadiradione |  |
|  |  | Bark Extract |  |  |  |  |  |
| 12. | Rajasekaran, 2008 | Leaves | Cold percolation method | Petroleum ether, dichloromethane, chloroform, ethanol and  aqueous extract | - | Ethanolic and  dichloromethane leaf extracts of *A. indica* were found to be more active towards the bacterial species |  |
| 13. | Manikandan *et al.*, 2012 | Leaf | Incubation | 70% Ethanol | HPTLC | - Nimbolide, - Nimonol, - 6-deacetyl - Nimbinene, - Quercetin | - By comparing densitogram peak areas |
|  |  |  |  |  | GCMS | - Nimbolide - Quercetin | - By Mass-selective   detection |
|  |  |  |  |  | LC-APCI-MS | - Nimbolicin, - Azadirachtin F - Tetratriacontane | - Identification was done by   comparing the mass spectra of samples with |

|  |  |  |  |  |  |  | molecular weight of compounds from earlier publications. |
| --- | --- | --- | --- | --- | --- | --- | --- |
| 14. | Arumugam *et al.*, 2015 | Leaves | Soxhlet extraction | The aqueous  and Ethanol 70% | Antifungal activity | - |  |
| 15. | Sidhu *et al.*, 2004 | Seed extract | - | Methanol | HPLC | Nimbin concentration ranged from  18.2 to 636.8 mg kg−1, whereas salanin as in the range of 45.4–1830.3 mg kg−1 kernel weight. | - The peaks were confirmed by comparing with standards. |

**References:**

1. Seriana, I., Akmal, M., Darusman, D., Wahyuni, S., Khairan, K., & Sugito, S. (2021). Phytochemicals characterizations of neem (*Azadirachta indica* A. Juss) leaves ethanolic extract: an important medicinal plant as male contraceptive candidate. *Rasayan Journal of Chemistry*, *14*(1), 343-350.
2. Alhaithloul, H. A., Alqahtani, M. M., Abdein, M. A., Ahmed, M. A., Hesham, A. E. L., Aljameeli, M. M., Al Mozini, R.N., Gharsan, F.N., Hussien, S.M., & El-Amier, Y. A. (2023). Rosemary and neem methanolic extract: antioxidant, cytotoxic, and larvicidal activities supported by chemical composition and molecular docking simulations. *Frontiers in Plant Science*, *14*, 1155698.
3. Almaghamsi, A. W., Gull, M., Jambi, E., Al-Hejin, A., Bataweel, N. M., & Qattan, S. Y. A. (2023). Antimicrobial Potential of *Azadirachta indica* (Neem) Leaves Extracts Against Various Pathogenic Bacteria. *Journal of Biochemical Technology*, *14*(2-2023), 117-124.
4. Arumugam, P. A., Mohamad, I., Salim, R., & Mohamed, Z. (2015). Antifungal Effect of Malaysian Neem Leaf Extract on Selected Fungal Species Causing Otomycosis in *In-vitro* Culture Medium. *Malaysian Journal of Medicine & Health Sciences*, *11*(2).
5. Dhillon, N., Garnaik, U. C., Yadav, D., Gupta, R., & Agarwal, S. (2024). Analysis of a therapeutic rich phytopharmaceutical compound of *Azadirachta indica*. *Spectroscopy Letters*, *57*(3), 122-127
6. Jahan, N., & Arju, S. N. (2022). A sustainable approach to study on antimicrobial and mosquito repellency properties of silk fabric dyed with neem (*Azadirachta indica*) leaves extractions. *Sustainability*, *14*(22), 15071.
7. Mayavel, A.., Krishnan, S., Abinaya, R., Esakkiammal, S., & Warrier, R. (2023). Variation in seed morphology and selected oil parameters of neem (*Azadirachta indica* A. Juss.) from different agroclimatic zones in Tamil Nadu, India. *Grasas Y Aceites*, *74*(4), e531.
8. Mehta, A., Jain, A., & Saxena, G. (2022). *In Vitro* Antibacterial Activity of Ethanolic Extract of Neem Leave (*Azadirachta indica* Linn) Against Clinical Isolates. *Pharmaceutical and Biomedical Research*, *8*(4), 301-310.
9. Mohammad, A. M., Shaker, A. M., Salem, O. M., Elmashay, A., & Hassani, R. (2024). Screening the larvicidal activity of ZnO, CuO nanoparticles, and neem seed oil extract against the full armyworm *Spodoptera frugiperda* (JE Smith) (Lepidoptera, Noctuidae). *Emirates Journal of Food and Agriculture*, *36*, 1-9.
10. Rajasekaran, C. (2008). Investigations on antibacterial activity of leaf extracts of *Azadirachta indica* A. Juss (Meliaceae): a traditional medicinal plant of India. *Ethnobotanical leaflets*, *2008*(1), 161.
11. Rajasekharan, R., Bahuleyan, A. K., Madhavan, A., Philip, E., Sindhu, R., Binod, P., Kumar Awasthi, M., & Pandey, A. (2023). Neem extract–blended nanocellulose derived from jackfruit peel for antibacterial packagings. *Environmental Science and Pollution Research*, *30*(4), 8977-8986.
12. Rangiah, K., Varalaxmi, B. A., & Gowda, M. (2016). UHPLC-MS/SRM method for quantification of neem metabolites from leaf extracts of Meliaceae family plants. Analytical Methods, 8(9), 2020-2031.
13. Sidhu, O. P., Kumar, V., & Behl, H. M. (2004). Variability in triterpenoids (nimbin and salanin) composition of neem among different provenances of India. *Industrial Crops and Products*, *19*(1), 69-75.
14. Sarkar, L., Oko, L., Gupta, S., Bubak, A. N., Das, B., Gupta, P., ... & Sarma, J. D. (2022). Azadirachta indica A. Juss bark extract and its Nimbin isomers restrict β-coronaviral infection and replication. Virology, 569, 13-28.
15. Manikandan, P., Ramalingam, S. M., Vinothini, G., Ramamurthi, V. P., Singh, I. P., Anandan, R., Gopalakrishnan, M., & Nagini, S. (2012). Investigation of the chemopreventive potential of neem leaf subfractions in the hamster buccal pouch model and phytochemical characterization. European Journal of Medicinal Chemistry, 56, 271-281.
